# Performance characteristics of a local triage tool and internationally validated tools among under-fives presenting to an urban emergency department in Tanzania

**DOI:** 10.1186/s12887-019-1417-7

**Published:** 2019-02-01

**Authors:** Nafsa R. Marombwa, Hendry R. Sawe, Upendo George, Said S. Kilindimo, Nanyori J. Lucumay, Kilalo M. Mjema, Juma A. Mfinanga, Ellen J. Weber

**Affiliations:** 1 Emergency Medicine Department, Muhimbili University of Health and Allied Science, P.O. Box 65001, Dar Es Salaam-Tanzania Dar es Salaam, Tanzania; 2 Emergency Medicine Department, Muhimbili National Hospital, Dar es Salaam, Tanzania; 3 Department of Emergency Medicine, University of California, San Francisco, California USA

**Keywords:** Triage, Emergency department, Triage scales, Africa, Tanzania

## Abstract

**Background:**

A number of region-specific validated triage systems exist; however very little is known about their performance in resource limited settings. We compare the local triage tool and internationally validated tools among under-fives presenting to an urban emergency department in Tanzania.

**Methodology:**

Prospective descriptive study of consecutive under-fives seen at Muhimbili National Hospital (MNH), ED between November 2017 to April 2018. Patients were triaged according to Local Triage System (LTS), and the information collected were used to assign acuities in the other triage scales: Canadian Triage and Acuity Scale (CTAS), Australasian Triage Scale (ATS), Manchester Triage Scale (MTS) and South African Triage Scale (SATS). Patients were then followed up to determine disposition and 24 h outcome. Sensitivity, specificity, positive and negative predictive values for admission and mortality were then calculated.

**Results:**

A total of 384 paediatric patients were enrolled, their median age was 17 months (IQR 7–36 months). Using LTS, 67(17.4%) patients were triaged in level one, 291(75.8%) level 2 and 26 (6.8%) in level 3 categories. Overall admission rate was 59.6% and at 24 h there were five deaths (1.3%). Using Level 1 in LTS, and Levels 1 and 2 in other systems, sensitivity and specificity for admission for all triage scales ranged between 27.1–28.4% and 95.4–98% respectively, (PPV 90.3–95.3%, NPV 47.1–47.4%). Sensitivity for mortality was 80% for LTS, and 100% for the other scales, while specificity was low, yielding a PPV for all scales between 6.9 and 8%.

**Conclusion:**

All triage scales showed poor ability to predict need for admission, however all triage scales except LTS predicted mortality. The test characteristics for the other scales were similar. Future studies should focus on determining the reliability and validity of each of these triage tools in our setting.

**Electronic supplementary material:**

The online version of this article (10.1186/s12887-019-1417-7) contains supplementary material, which is available to authorized users.

## Background

Triage is the process of determining the priority of a patient to receive medical care based on the urgency and severity of their condition. It involves categorising patients into different urgency levels according to their medical complaints, vital signs, symptoms and available resources so that the most acutely ill are seen first [1, 2].

The most widely used triage scores are the Manchester Triage Score, the Australian Triage Score, the Canadian Triage Score and the Emergency Severity Index, all of which were developed in high income countries and categorize patients into five levels of acuity [3, 4]. Emergency Severity Index (ESI) differs from other triage scales in that, it determines the patient’s urgency based on their presentation and predicts the resource utilization as well as estimating resource utilisation for lower acuity categories [5].

The first triage system developed in Africa was the Cape Triage Score (CTS), and was validated in 2006. It has now been widely adopted throughout South Africa as the South African Triage Scale (SATS) [5, 6]. Unlike ATS, MTS, ESI and CTAS, this system has four levels [5, 7]. It uses triage early warning scores (TEWS) to predict the acuity level [8].

Triaging in paediatrics has been challenging due to several factors including the need for special communication skills and different response to physiological stressors such as dehydration and infections [9]. To overcome this, several triage systems have been adapted, developed or modified to improve the triage of children [10]. The ATS, CTAS and ESI all have paediatric modifiers to assist in using the same tool for both adults and children. MTS has complaint specific flow charts for children. SATS has a separate paediatric SATS scale as there are no modifiers within SATS for children [11–14].

To date there are few fully developed emergency departments in Tanzania; the Emergency Medicine Department of Muhimbili National Hospital (EMD-MNH) was the first and opened in 2010. The triage system used in this department has been adopted from ESI and modified to suit our population whereby patients are categorized as emergency, priority or queue, based on their presenting complaint, symptoms and signs [15]. The other triage scales (MTS, ATS, CTAS and SATS) have never been tested in this population. It is not known whether the current system of triage is optimal, or whether one of the existing validated scales should be used in LMIC settings such as ours.

## Methods

### Study design

This was a prospective descriptive study of a consecutive sample of paediatric patients below the age of five years with non-traumatic medical complaints presenting to the EMD-MNH from November 2017 to April 2018.

### Study setting

MNH is a national referral government hospital with 1500 beds, and attending 1500 outpatients’ everyday. It is located in Ilala district, Dar es Salaam City, Tanzania. The EMD was opened in 2010, and serves an annual average of 60,000 patients who are referred from across Tanzania. The hospital has no formal admission policy, the process of admission is based on combination of providers clinical gestalt (on severity of illness) and disease specific guidelines. About 25% of patients seen in this department are paediatric age with 75% presenting with non-traumatic medical conditions [16]. There is no validated triage tool that is used for triage of patients in the EMD.

### Participants

Paediatric patients below the age of five years with non-traumatic medical complaints presenting to the EMD-MNH were eligible. Patients arriving in respiratory or cardiac arrest, returning for follow up, or who had a history of trauma within two weeks were excluded. Consent for participation in the study was requested from the adult accompanying the child.

### Samples size

Our study population was paediatric patients below the age of five years, and we estimated the minimum sample size of 372 assuming a 59% admission rate, based on the local EMD data for the year before this study. This sample size allowed a precise comparison of proportions of triage levels by each triage scale.

### Study protocol

A research assistant (RA) was scheduled in the department every other day for 8–12 h during the study period alternating between day and nights. Eligible patients were consecutively enrolled after written informed consent was obtained. The RA recorded initial triage information including chief complaint, vital signs and acuity assigned by the triage nurse. During the triage encounter, the RA calculated triage acuity for each patient, using each of the following- MTS, CTAS with paediatric modifiers, ATS with paediatric modifiers and paediatric SATS (Additional file 1). Subsequent to triage, the RA recorded investigations done, ED diagnosis and disposition from review of the electronic medical record while the patient was in the ED. (Wellsoft Version 11 Corporation, Somerset, NJ, USA). Demographics and other necessary information for triage were also obtained from mother / guardian through interview. All children were followed up. For those discharged, we contacted the parents/ guardians by mobile phone (a call was made 3 times on three different days), and for those admitted; we followed the patient to the specific wards in order to obtain their 24-h outcome.

Outcomes: The outcomes of interest were the sensitivity, specificity, PPV and NPV of the triage acuity on each scale for predicting admission and mortality.

### Data analysis

Data were entered into SPSS software (V23) and analysed. Descriptive statistics (counts, percentages, median, quartiles,) were obtained for demographic characteristics, distribution of patients in different triage categories by each triage scale, and the proportions of admissions and deaths within each triage acuity. To assess test characteristics (sensitivity, specificity, PPV, NPV) for prediction of admission and 24-h mortality of each scale, we created 2 groups from each triage scale: For ATS, CTAS and MTS: high acuity Level 1 and 2, low acuity 3–5 (to level 4 in SATS), and for LTS, high acuity level 1 and low acuity level 2 and 3.

## Results

### Demographic characteristics

**Fig. 1:**
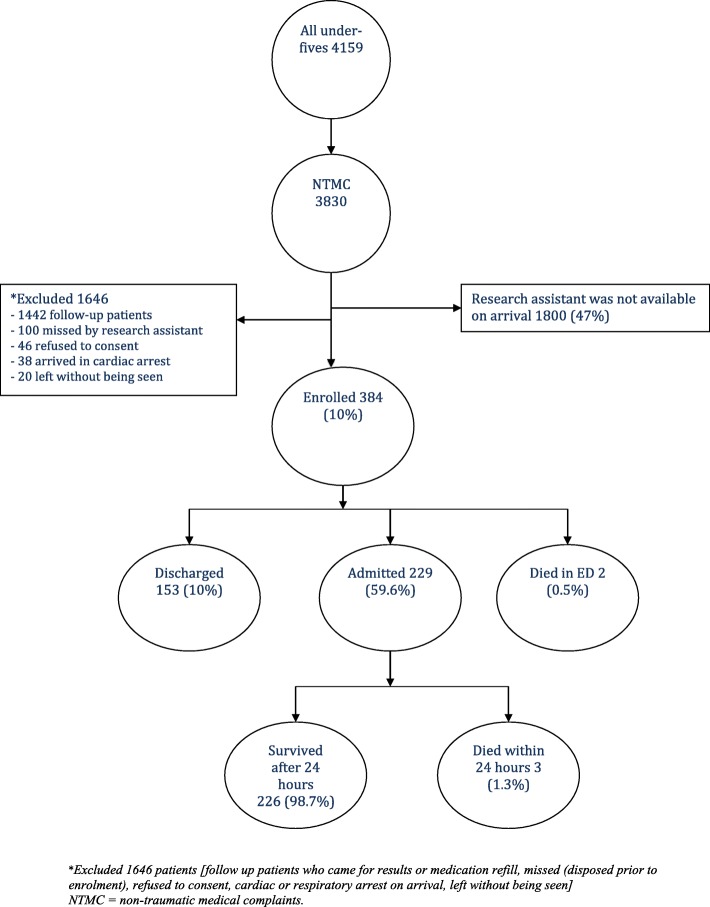
Patients enrollment flow diagram

We enrolled and followed up 384 out 2030 under fives with non-traumatic complaints who presented to EMD during times when research assistant was available. Figure 1.

Of the 384 patients that were followed up, the median age was 17 months (IQR 7–36 months) 211 (54.9%) were male. In the local triage system, 67 (17.4%) were triaged as emergency, 291 (75.8%) in priority group and 26 (6.8%) in queue group. Overall, 178 (46.4%) of patients were referred from other hospitals. Table 1.

**Table 1:** Demographics characteristics of children presented to ED with NTMC

Characteristics	*N* = 384
Median age in months (IQR)	17 (7–36)
Sex – male	211 (54.9)
Referral status	n (%)
Referred	178 (46.4)
Not referred	206 (53.6)
Co-morbidities	*N* = 25
Sickle cell disease	10 (40)
Congenital heart disease	13 (52)
HIV	2 (8)
Admission rate	229 (59.6%)
ED outcome	n (%)
Admitted	229 (59.6)
Discharged	153 (39.4)
Died	2 (0.5)
Local triage scale	n (%)
Emergency (level 1)	68 (17.4)
Priority (level 2)	291 (75.8)
Queue (level 3)	26 (6.8)

KEY: HIV –Human Immunodeficiency Virus

### Triage assignment and outcomes

Among the enrolled patients, 229 (59.6%) were admitted, 153 (39.9%) discharged and 2 (0.5%) died at ED. The proportion of patients admitted was highest in the high acuity groups, and decreased with level of acuity in all scales; the proportion of discharges was lowest in the high acuity groups and increased with lower acuity assignments. Five patients (1.45%) died within 24 h, including the 2 ED deaths (both were haemodynamically unstable and they were triaged as level 1 in all triage scales). The MTS, ATS, CTAS and SATS categorized all of these patients as acuity 1 or 2, while LTS categorized 4 patients as emergent and 1 as priority. Table 2.

**Table 2:** Relationship of triage scales with admission, discharge and mortality

Triage system	Triage levels	Emergency Department Disposition		
		Admitted	Discharged	Died at ED
LTS	1	62(92.5%)	3(4.5%)	2(3%)
	2	154(52.9%)	137(47.1%)	–
	3	13(50%)	13(50%)	–
MTS	1	33(91.4%)	1(2.9%)	2(5.7%)
	2	32(88.9%)	4(11.1%)	–
	3	35(81.4%)	8(18.6%)	–
	4	79(56.4%)	61(43.6%)	–
	5	50(38.8%)	79(61.2%)	
ATS	1	22(91.7%)	1(4.2%)	1(4.2%)
	2	43(89.6%)	4(8.3%)	1(2.1%)
	3	36(76.6%)	11(23.4%)	–
	4	52(60.5%)	34(39.5%)	–
	5	76(42.5%)	103(57.5%)	–
CTS	1	26(92.9%)	1 (3.6%)	1 (3.6%)
	2	37(88.1%)	4(9.5%)	1(2.4%)
	3	54(77.1%)	16(22.9%)	–
	4	35(47.3%)	39(52.7%)	–
	5	77(45.3%)	93(54.7%)	–
SATS	1	43(95.6%)	1(2.2%)	1(2.2%)
	2	22(75.9%)	6(20.7%)	1(3.4%)
	3	48(73.8%)	17(26.2%)	–
	4	116(47.3%)	129(52.7%)	–

**Fig. 2:**
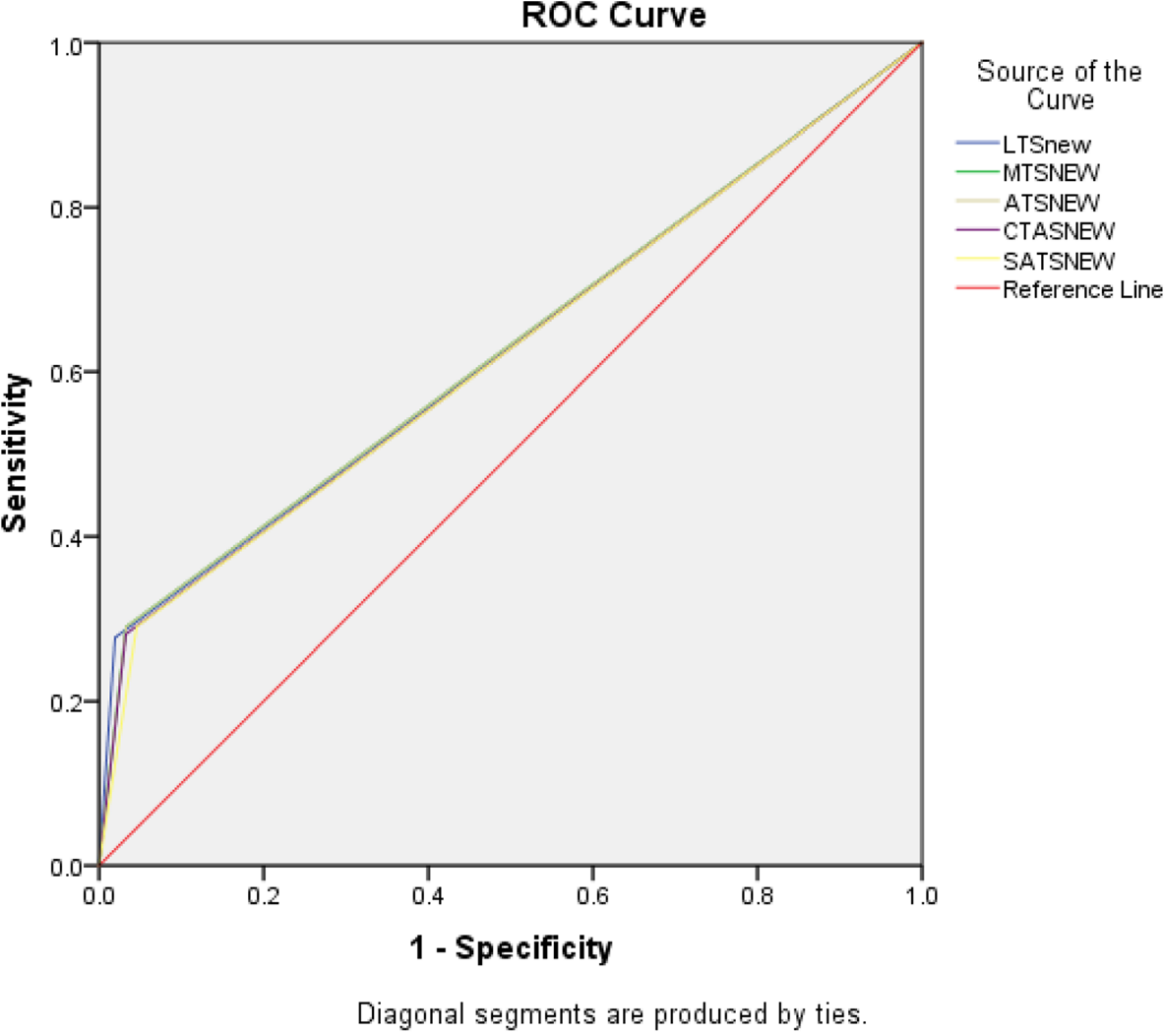
The ROC curve for admission

The area under the ROC (AUROC) curve for the outcome of admission for all triage scales ranged between 0.62–0.63 (Fig. 2). Sensitivity of high acuity assignment for admission ranged between 27.1 and 29.4%, while specificity ranged from 95.4 to 98.0%. Results were similar among all triage scales. Table 3.

**Table 3:** Performance of triage scales in predicting Admission

Admission prediction	LTS	MTS	ATS	CTAS	SATS
Sensitivity (95% CI)	27.1% (22–33%)	28.4% (23–35%)	28.4% (23–35%)	27.5% (22–34%)	28.4% (23–35%)
Specificity (95% CI)	98% (94–100%)	96.7% (92–99%)	96.7% (92–99%)	96.7% (92–99%)	95.4% (90–98%)
PPV (95% CI)	95.4% (86–99%)	92.9% (83–97%)	92.9% (83–97%)	92.6% (83–97%)	90.3% (80–96%)
NPV (95% CI)	47.3% (42–53%)	47.4% (42–53%)	47.4% (42–53%)	47.1% (42–53%)	47.1% (42–53%)

**Fig. 3:**
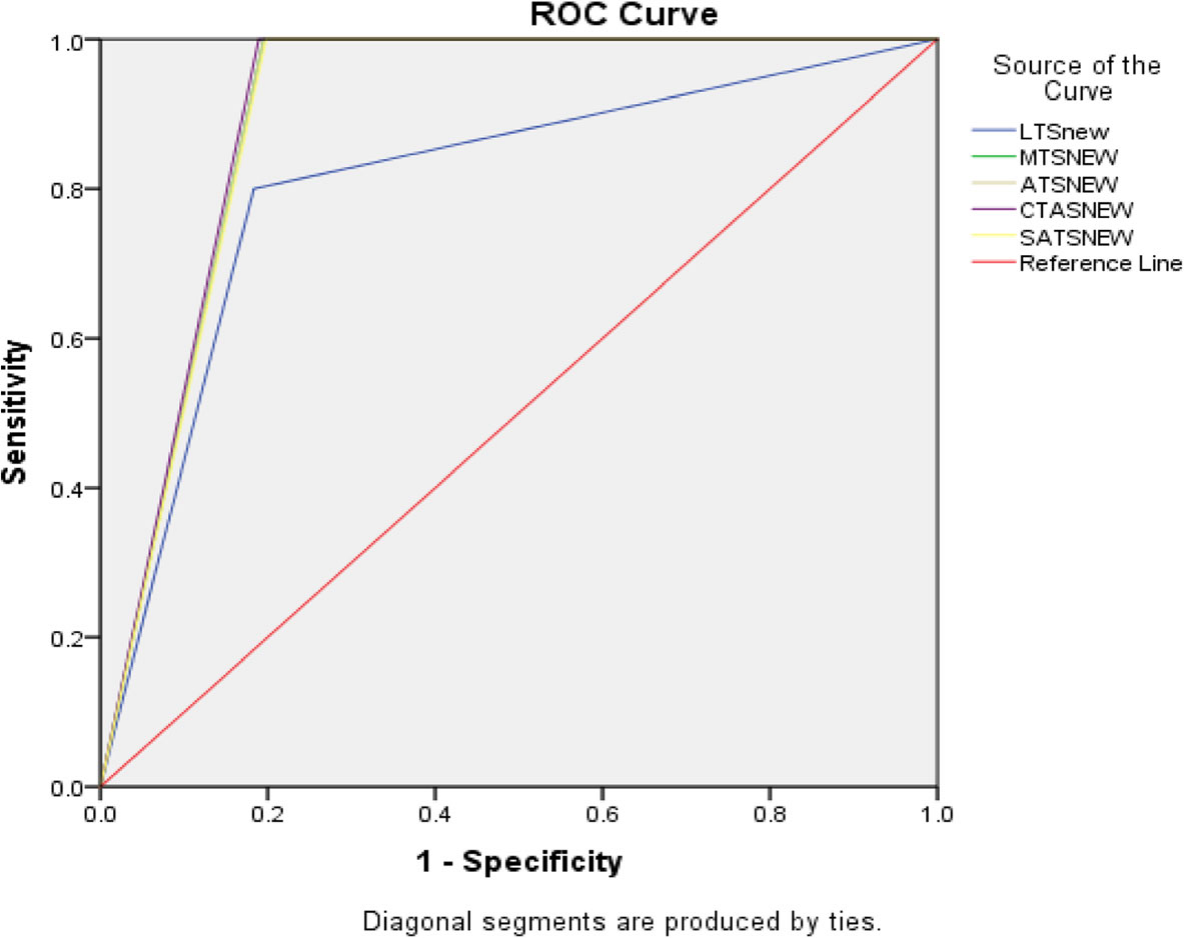
The ROC curve for 24-h mortality

With regard to ability to predict 24 h mortality, 41 patients were excluded due to missing data on 24 h outcomes. The AUROC curve for mortality ranged between 0.90–0.91 for all scales except for the LTs, where the AUROC was 0.81 (Fig. 3). The sensitivity for mortality was 80% for LTS and 100% for the other scales; NPV was 99.6%. (98–100%) for LTS and 100% (98–100%) for all other scales. Table 4.

**Table 4:** Performance of triage scales in predicting 24 h Mortality

Prediction of 24 h mortality	LTS	MTS	ATS	CTAS	SATS
Sensitivity (95% CI)	80% (30–99%)	100% (46–100%)	100% (46–100%)	100% (46–100%)	100% (46–100%)
Specificity (95% CI)	81.4% (77–85%)	80.2% (76–84%)	80.2% (76–84%)	80.8% (76–85%)	79.9% (75–84%)
PPV (95% CI)	6% (2–15%)	6.9% (3–16%)	6.9% (3–16%)	7.1% (3–17%)	6.8% (3–16%)
NPV (95% CI)	99.6% (98–100%)	100% (98–100%)	100% (98–100%)	100% (98–100%)	100% (98–100%)

## Discussion

Triage systems prioritize the care of ED patients according to the severity of their illness. In this study, patients were assigned acuity levels using five triage scales based on their initial presentation to the ED. The local triage system (LTS) triaged more patients 67(17.4%) in level 1 compared to MTS, CTAS, ATS and SATS. However this difference is likely due to the differences in number of acuity levels; LTS has 3 levels unlike MTS, CTAS and ATS, which have 5 levels, and SATS that has 4 levels.

Because there is no “gold standard” for acuity, many prior studies have used proxy outcomes to determine how well a triage system works [17]. In our study, we used admission rates and mortality rates, as have others. Using these proxy outcomes, the ideal triage tool should be able to correctly identify those patients who will require admission and assign them to a high acuity while those who will be discharged would be assigned to the least acute categories. Similarly, the ideal scale would classify those with a high risk of mortality into the highest acuity levels. In this study, we found that the performance of all five scales for disposition was similar: the proportion of patients discharged decreased with the level of urgency. However, performance of the scales was different with regard to mortality. While the MTS, ATS, CTAS and paediatric SATS classified patients who died as either Level 1 or 2, the LTS missed one patient, classifying them as “priority” rather than “emergency.”

In predicting need for admission, all triage scales were found to have low sensitivity (< 50%) and high specificity. These findings differ from those found in a study done in Thailand where MTS and CTAS had higher sensitivity and specificity compared to those found in our study [18]. The low sensitivity for admission observed among all triage scales in this study means many patients assigned low acuities were nevertheless admitted (false negatives). This is due to the hospital admission policies where patients may be admitted for reasons other rather than acuity. (Example: patients referred from other regions other than Dar es Salaam have to be admitted as they have no other place to stay.) Our ED’s overall admission rate for patients under 5 is 59.6%. In a validation study for the revised pediatric SATS scale, the proportion of patients discharged rose from 27.2% among emergency category to 95.3% in non-urgent patients, whereas even in the lowest acuity category in our study, more than 50% of patients were admitted [19].

In predicting mortality, LTS showed least ability to predict mortality (sensitivity of 80%) compared to MTS, ATS, CTAS and SATS (sensitivity of 100%, specificity 100%). The high sensitivity for mortality among these four scales is likely due to the overall low mortality rate in the population studied. The ED accepts non-emergency patients who come as insured / private patients and also those who come for elective admission or specialist referral. These are mostly stable patients, and can easily be identified.

## Limitations

Our study was a single centre study, which could limit the generalizability of the results. Also, lack of a gold standard for acuity meant that proxy outcomes were used for test characteristics. Decisions on admission and discharge may be affected by social reasons other than clinical indications making this outcome less useful in our hospital.

## Conclusion

Among under-fives presenting to an ED of an urban tertiary hospital in Tanzania, all triage scales showed poor ability to predict need for admission. All triage scales except LTS predicted mortality and test characteristics among the other scales were similar. Future studies should focus on determining the reliability and validity of each of these triage tools in our setting.

## Additional file

Additional file 1: Summary of differences in triage scales (DOC 31 kb)

## Abbreviations

ATS: Australasian triage scale
CTAS: Canadian triage and acuity scale
EMD: Emergency medicine department
ESI: Emergency severity index
ETAT: Emergency triage, assessment and treatment
HIV: Human immunodeficiency virus
ITS: Ipswich triage scale
LTS: Local triage scale
MNH: Muhimbili National Hospital
MTS: Manchester triage scale
MUHAS: Muhimbili University of Health and Allied Science
PedCTAS: Paediatric Canadian triage and acuity scale
SATS: South African Triage Scale
TEWS: Triage early warning score
TS: Triage scale
WHO: World health organisation
