# Supplementary material for: Performance characteristics of a local triage tool and internationally validated tools among under-fives presenting to an urban emergency department in Tanzania
Source: BMC Pediatr. 2019 Feb 1;19:44. doi: 10.1186/s12887-019-1417-7 (PMC6357459; doi:10.1186/s12887-019-1417-7)
Supplement: Supplementary file 1 — Summary of differences in triage scales (DOC 31 kb) [file 12887_2019_1417_MOESM1_ESM.doc]

Summary of differences in triage scales

| **Triage scale** | **Country** | **Triage levels** | **Recommended waiting time (minutes)** | | | | |
| --- | --- | --- | --- | --- | --- | --- | --- |
|  |  |  | Level 1 | Level 2 | Level 3 | Level 4 | Level 5 |
| LTS | Tanzania | 3 | Immediate | Not specified | Not specified | N/A | N/A |
| MTS | United Kingdom | 5 | Immediate | 10 | 60 | 120 | 240 |
| CTAS | Canada | 5 | Immediate | 10 | 30 | 60 | 120 |
| ATS | Australia | 5 | Immediate | 10 | 30 | 60 | 120 |
| SATS | South Africa | 4 | Immediate | <10 | <60 | <240 | N/A |
